# Socio-demographic Characteristics and Psychiatric Disorders of Patients Aged 60 and Over Admitted to the Psychiatry Department at Razi Hospital, Tunisia

**DOI:** 10.1192/j.eurpsy.2025.1723

**Published:** 2025-08-26

**Authors:** S. Aouadi, H. Ghabi, Y. Rebei, M. Karoui, H. Nefzi, R. Kammoun, F. Ellouze

**Affiliations:** 1 Psychiatry G, Razi Hospital; 2 Faculty of Medecine of Tunis, El Manar university, Tunis, Tunisia

## Abstract

**Introduction:**

Psychiatric issues are prevalent among the elderly, and as people age, they often face both medical and psychiatric comorbidities, creating significant challenges. However, there is a notable lack of literature on inpatient studies focused on this demographic.

**Objectives:**

This study aims to assess the socio-demographic profile of patients over the age of 60 admitted to a psychiatric unit at Razi Hospital, Tunisia, while identifying the prevalence of various psychiatric disorders.

**Methods:**

This was a retrospective and descriptive study. It included patients aged 60 and above who were first-time psychiatric admissions at the El Jazzar department of CHU Razi between 2020 and 2024. Data were collected from medical records, covering variables such as gender, age, year of admission, marital status, education level, occupation, socioeconomic status, medical history, prior treatments, diagnoses, admission type, and discharge treatments.

**Results:**

In this study, we reviewed 40 case files. The analysis revealed that the majority of patients were male (80%), with most falling within the 60-70 age range. The majority were married (65%), and educational attainment was typically low, with 60% having a primary education level or below. Retirees accounted for 65% of the sample. Regarding comorbidities, 70% of the patients had both medical and psychiatric histories. The primary reasons for admission included behavioral disorders, delusional syndromes, and depressive disorders. Prior to admission, treatments mainly consisted of mood stabilizers and first- or second-generation antipsychotics. The most frequent mode of admission was hospitalization at the request of a third party (HDT), followed by involuntary hospitalization (HO). During hospitalization, prescribed treatments included antipsychotics (both atypical and typical), benzodiazepines, and antidepressants. No significant adverse drug reactions were reported. Dementia, recurrent depression, and psychotic relapse in the context of schizophrenia were the most common diagnoses.

**Conclusions:**

This study provides valuable insights into the socio-demographic and clinical profile of patients over 60 admitted to psychiatry at Razi Hospital, highlighting key trends in mental health and treatment approaches. The frequent presence of somatic comorbidities emphasizes the need for a multidisciplinary therapeutic approach. These findings underscore the importance of specialized treatment for the complex needs of this elderly population.

**Disclosure of Interest:**

None Declared

